# *Lilioceris groehni* sp. n.: the first authentic species of Criocerinae (Coleoptera, Chrysomelidae) from Baltic amber

**DOI:** 10.3897/zookeys.618.10085

**Published:** 2016-09-19

**Authors:** Andris Bukejs, Michael Schmitt

**Affiliations:** 1 Institute of Life Sciences and Technologies, Daugavpils University, Vienības Str. 13, Daugavpils, LV-5401, Latvia; 2 Ernst-Moritz-Arndt-Universität, Allgemeine & Systematische Zoologie, Soldmannstr. 14, 17489 Greifswald, Germany

**Keywords:** Taxonomy, palaeontology, shining leaf beetles, new species, Crioceris pristiana, fossil resin, Tertiary, Eocene

## Abstract

Based on a single well-preserved specimen from Eocene Baltic amber, *Lilioceris groehni* **sp. n.** is described and illustrated using phase-contrast X-ray microtomography. It is the first described species of Criocerinae (Coleoptera: Chrysomelidae) from Baltic amber. A check-list of fossil Criocerinae is provided. Placement of *Crioceris pristiana* (Germar, 1813) is discussed, this species is removed from Criocerinae and placed in Coleoptera *incertae sedis*.

## Introduction

The subfamily Criocerinae (shining leaf beetles) contains ca. 1500 extant species (Schmitt 1996) in 20 genera (Seeno and Wilcox 1982), of which 211 species in 6 genera are recorded from the Palaearctic region (Schmitt 2010). The genus *Lilioceris* Reitter, 1913 comprises ca. 170 extant species distributed over the temperate, subtropical and tropical regions of the Palaearctis, Orientalis, Aethiopis including Madagascar, and the Australis, and was introduced to North America by man (Monrós 1960, plus records from the Zoological Record up to present as taken from the Index to Organism Names).

Shining leaf beetles are rarely represented in fossil material and especially in Baltic amber (Table 1). Two fossil species from Baltic amber were mistakenly described within Criocerinae. *Electrolema baltica* Schaufuss, 1892 was described as member of Criocerinae but later transferred to Hispinae (Korschefsky 1939). According to modern classification (Staines 2012), it is placed in Gonophorini Chapuis, 1875 within Cassidinae. Another species, *Crioceris pristina* (Germar, 1813) originally described as *Criocerina* (Germar 1813) was mentioned within Criocerinae (e.g. Giebel 1856a, 1856b; Spahr 1981; Santiago-Blay 1994). In our opinion it is not a member of the Criocerinae (see Discussion).

**Table 1.** Check-list of records of fossil and sub-fossil Criocerinae.

Taxon	References	Fossil Type	Locality	Age
Criocerinae	Bachofen-Echt 1949; Handlirsch 1925; Spahr 1981	Baltic amber	Kaliningrad region (Russia)	37.2–33.9 Ma
Criocerinae	Hayashi et al. 2002	poorly lithified peat	Mizozono Formation, Yoshimatsu-cho, Kagoshima Prefecture (Japan)	0.1–0.0 Ma
*Crioceridea dubia*	Wickham 1912, 1913, 1914a, 1920; Santiago-Blay 1994	lacustrine shale	Florissant, Colorado (USA)	37.2–33.9 Ma
*Crioceris margarum*	Oustalet 1874; Handlirsch 1908; Théobald 1937; Santiago-Blay 1994	lacustrine shale	Aix-en-Provence (France)	28.4–23.0 Ma
*Crioceris vetusta*	Heer 1865 (*Lema*); Handlirsch 1908 (*Lema*); Cockerell 1921	lacustrine shale	Oeningen (Germany)	12.7–11.6 Ma
*Crioceris* sp.	Hope 1836; Giebel 1856a; Menge 1856; Scudder 1885, 1886, 1891; Handlirsch 1908; Klebs 1910; Bachofen-Echt 1949; Spahr 1891	Baltic amber	Kaliningrad region (Russia)	37.2–33.9 Ma
*Lema evanescens*	Wickham 1910, 1913, 1914a, 1920	lacustrine shale	Florissant, Colorado (USA)	37.2–33.9 Ma
*Lema fortior*	Wickham 1914a, 1920	lacustrine shale	Florissant, Colorado (USA)	37.2–33.9 Ma
*Lema lesquereuxi*	Wickham 1914b, 1920	lacustrine shale	Florissant, Colorado (USA)	37.2–33.9 Ma
*Lema tumulata*	Heyden and Heyden 1865; Handlirsch 1908	terrestrial siliciclastic	Salzhausen (Germany)	15.9–11.6 Ma
*Lema* sp.	Scudder 1885, 1886, 1891; Helm 1896; Handlirsch 1908; Larsson 1978; Spahr 1981; Poinar 1999	Baltic amber	Kaliningrad region (Russia)	37.2–33.9 Ma
*Lema* sp.	Pearson 1962	lacustrine shale	West Cumberland (England)	0.1–0.0 Ma
(?)*Lema* sp.	Kiselev and Nazarov 2009	unlithified siliciclastic sediments	Achchagyai-Allaikha Yana–Indigirka Lowland, nord-east Siberia (Russia)	0.1–0.0 Ma
*Lilioceris groehni*	present paper	Baltic amber	Kaliningrad region (Russia)	37.2–33.9 Ma
Coleoptera *incertae sedis*				
*Crioceris pristina*	Germar 1813 (*Criocerina*); Giebel 1856a, 1856b; Schlechtendal (*Criocerina*); Handlirsch 1908; Spahr 1981; Santiago-Blay 1994; Poinar 1999	Baltic amber	Kaliningrad region (Russia)	37.2–33.9 Ma

*Crioceris* sp. and *Lema* sp. were mentioned from Eocene Baltic amber without detailed species descriptions (Hope 1836; Giebel 1856; Menge 1856; Scudder 1885, 1886, 1891; Handlirsch 1908; Klebs 1910; Bachofen-Echt 1949; Larsson 1978; Spahr 1981; Santiago-Blay 1994; Poinar 1999). In the current paper, the first extinct species of Criocerinae from Baltic amber is described, figured, and compared with extant species using phase-contrast X-ray microtomography.

## Material and methods

The specimen is included in an amber piece that was polished by hand and facetted on their sides, allowing improved views of the included specimens. The material examined is deposited in the collection of the Geological-Palaeontological Institute of the University of Hamburg, Germany [GPIH], as part of the collection of Carsten Gröhn.

Observations were made using a Nikon SMZ 745T stereomicroscope. Photographs were taken using a Canon EOS 70D with a 100 mm macro lens, and a Canon EOS 5D with the Canon MP E 65 mm macro lens in a visionary digital bk plus lab system by Dun Inc. The microCT-images were produced by means of an Xradia Micro XCT-200 (Carl Zeiss X-ray Microscopy Inc.), using the 4x object lens units, at 30kV and 4W, with a pixel size of 5.36 µm. Tomography projections were reconstructed using the reconstruction software provided by XRadia. Volume rendering of image stacks was performed by using Amira 5.6.0 (FEI Visualization Science Group, Burlington, USA) using the “Volren” or “Voltex” function.

## Systematic Palaeontology

### Chrysomelidae Latreille, 1802
Criocerinae Latreille, 1804
Criocerini Latreille, 1804
*Lilioceris* Reitter, 1913

The specimen considered here was assigned to the family Chrysomelidae based on the pseudoteramerous tarsi and the lack of a rostrum and of antennae not inserted on pronounced tubercles, to the subfamily Criocerinae because the prothorax does not bear side borders and the frons has distinct diverging grooves behind the antennal insertions, and to the genus *Lilioceris* based on (1) free tarsal claws and (2) divided vertex separated from the neck by a dorsal constriction.

#### Lilioceris groehni sp. n.

Taxon classificationAnimaliaColeopteraChrysomelidae

http://zoobank.org/FD228756-DF55-47E3-BDE4-F0D3937A8A1E

Figs 1–2 , 3–5

##### Type material.

**Figures 1–2.**
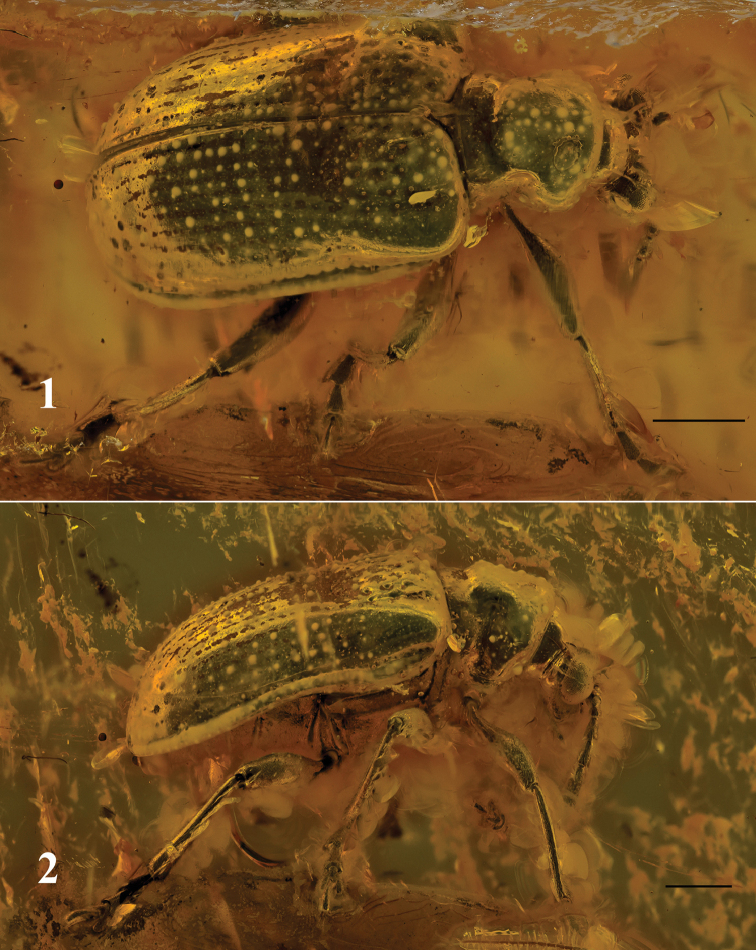
*Lilioceris groehni* sp. n., holotype: **1** habitus, dorsal view **2** habitus, lateral view. Scale bars: 1 mm.

Holotype: Nr. “C 8130” [GPIH]; female. A rather complete beetle (missing apical antennomere of left antenna, and tarsomeres 4 and 5 of left meso- and metatarsi) is included in a small, transparent yellow amber piece (length about 20 mm, width 12 mm, and maximum thickness 5 mm). Syninclusions: one specimen of Nematocera (Diptera), and few stellate Fagaceae trichomes (Figs 1 and 2).

##### Type strata.

Baltic amber, mid-Eocene to Upper Eocene.

##### Type locality.

Yantarny settlement (formerly Palmnicken), Sambian (Samland) Peninsula, the Kaliningrad region, Russia.

##### Differential diagnosis.

Head, body, and elytra of *Lilioceris groehni* sp. n. appear unicolorous black and thus similar to the extant species *Lilioceris hitam* Mohamedsaid, 1990 from Borneo, which differs from the new species in (1) the shape of the pronotum (distinctly longer than wide with its constriction at the middle), (2) metaventrite glabrous in the middle, (3) pubescent scutellum, (4) impunctate elytra (with few moderately large punctures at base only), (5) vertex with sparse pubescence, (6) a distinct conical neck between head and pronotum, and (7) a larger body (10 mm).

Additionally, the extant species *Lilioceris lilii* Scopoli, 1763 and *Lilioceris merdigera* Linnaeus, 1758 from Baltic region differ from *Lilioceris groehni* sp. n. in having (1) a pronotum with a longitudinal row of punctures medially, (2) metaventrite, metepisternum and ventrites of abdomen almost glabrous or with very sparce pubescence, and (3) pronotum and elytra rufous to red.

##### Description.

Holotype. Body length 7.1 mm, maximum width 4.1 mm; elongate, subparallel, moderately convex dorsally and ventrally, unicolorous black, glabrous dorsally.

**Figures 3–5.**
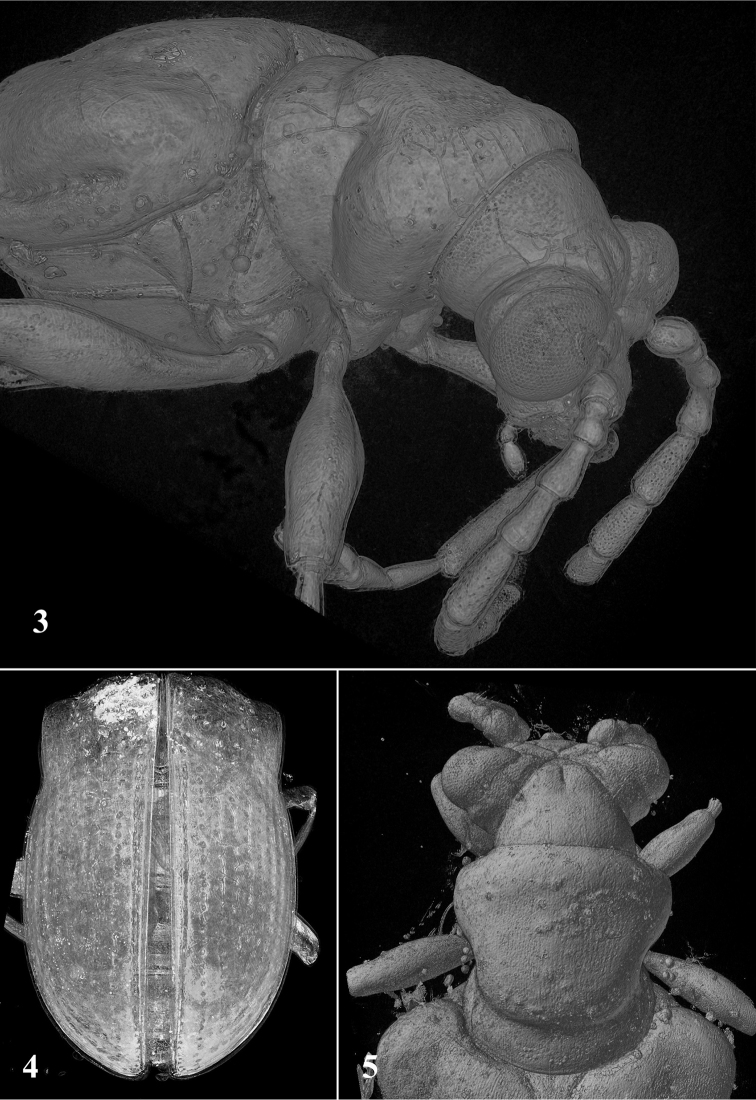
*Lilioceris groehni* sp. n., holotype, microCT images: **3** habitus, fronto-lateral view, showing the pronounced arcuate constriction behind the disk of the pronotum **4** elytra, dorsal view **5** details of head and prothorax, dorsal view. Not reproduced to the same scale.

Head hypognathous, transverse, widest across eyes, together with eyes nearly as wide as pronotum, strongly constricted behind the eyes forming a neck (Fig. 3); shiny, hairless and without distinct punctures dorsally. Compound eyes large, strongly convex, deeply and acutely notched at antennal insertions; distance between eyes nearly as wide as transverse diameter of one eye. Frontal grooves deep, crossed forming X. Vertex convex, hairless, with median longitudinal groove. Genae large, with sparse pubescence. Antennae poorly visible because of a beetle location in amber piece. Antennae robust, covered with fine pubescence, moderately long, extending nearly to basal one-fourth of elytra, slightly widened apically; antennomere 2 shortest, about 0.4 times as long as antennomere 3, antennomere 4 sligthly longer than antennomere 3, antennomeres 5–7 subeqal in length, antennomere 5 about 1.2 times as long as antennomere 4.

Pronotum nearly as long as wide, deeply constricted medially, distinctly narrower than elytra, widest in anterior one-third; impunctate, shiny; disc flattened, with an arcuate transverse depression subbasally (Figs 3 and 4). Anterior margin straight medially; posterior margin convex; lateral margins rounded anteriorly and strongly constricted just behind middle; all margins not bordered. Anterior and posterior angles obtusely rounded.

Scutellum large, triangular; apparently hairless and impunctate. Elytra subparallel, widest in the middle, about 1.5 times as long as wide; humeri prominent. Elytral punctures small and dense (in basal one-third deeper), arraged in rows; scutellar row present, short; intervals flat, only at apices weakly convex.

Metaventrite with sparse, fine pubescence; metepisternum and metepimeron densely covered with fine, short, semierect pubescence. Abdomen with sparse, fine pubescence.

Legs moderately long, covered with fine pubescence. Femora spindle-shaped; tibiae slightly curved, dilated apically. Tarsi long, about 0.7 times as long as tibia; metatarsomeres 1–2 subequal in length, distinctly dilated apically, metatarsomere 3 deeply bilobed, metatarsomere 4 subcilyndrical, narrow. Tarsal claws free, not fused at base.

The interior of the abdomen does not contain any identifiable structure, as revealed by the microCT-analysis. No traces of an aedeagus could be found, and none of the smaller particles – all covered with homogeneous material – could be addressed as the spermatheca.

##### Derivatio nominis.

This new species is named after Carsten Gröhn (Glinde, Germany) – he enabled us to study this specimen.

## Discussion

The specimen of *Lilioceris groehni* sp. n. appears externally complete. However, the fact that we found no traces of internal structures in the abdomen, especially of an aedeagus, does most probably mean that (1) the specimen was a female, and (2) that it remained openly accessible for scavengers and/or detritivores before it was covered by resin. This could also provide a possible reason for its black appearance as the dead individual might have been exposed to humic acids before being fossilised. If this should be the case, the live animal had most probably a habitus similar to the extant lily beetles. Actually, *Lilioceris groehni* sp. n. is hardly distinguishable from extant *Lilioceris*-species. In this respect, the new species is quite normal. Hennig (1966) wrote that it is „a long known fact” [„*eine altbekannte Tatsache*”] that the morphological differences between fossils from Baltic amber and their extant relatives are only minute”.

Santiago-Blay (1994) mistakenly listed two fossil species as members of Criocerinae: *Lema pervetusta* Cockerell, 1921 and *Lema pulchella* Förster, 1891. *Lema pervetusta* was described from Bridgerian lacustrine shale (Eocene, 50.3–48.6 Ma) of the Green River Formation of Colorado, USA (Cockerell 1921), but according to Linsley (1942) this species belongs to the longhorn beetle genus *Clytus* Laicharting, 1784 (Cerambycidae). *Lema pulchella* was described from Oligocene lacustrine (33.9–28.4 Ma) of Riedisheim, Mulhouse, France (Förster 1891), but according to Théobald (1937) it belongs to the weevil genus *Phyllobius* Germar, 1824 (Curculionidae).

Few Quaternary sub-fossil records contain specimens of the extant species: *Lema cyanella* (Linnaeus, 1758) from La Taphanel, Massif Central, France (Ponel and Coope 1990); *Lema trilinea* White, 1981 from late Quaternary Kaetan Cave, Colorado Plateau, Colorado, USA (Elias and Van Devender 1992); and *Oulema obscura* (Stephens, 1831) from the Holocene of Belarus (Nazarov 1984). These records are not mentioned in the current list (Table 1). None of the records of “Criocerinae”, “*Crioceris* sp.”, or “*Lema* sp:” from Baltic amber listed in Table 1 can be assigned to a certain species of shining leaf beetles.

Germar (1813) described *Criocerina pristina* from Baltic amber. Later this species was mentioned as *Crioceris pristina* (Germar, 1813) within Criocerinae (e.g. Giebel 1856a, 1856b; Spahr 1981; Santiago-Blay 1994). The correct subfamily and family placement of *Crioceris pristina* is doubtful in our opinion. According to the original description (Germar 1813: 14), this fossil species has antennae with a club (similar as in members of the genus *Anobium*) ["... *Die Fühler von etwas mehr als halber Körperlänge, roth, and der Spitze dunkler, das erste Glied kurz und dick, vor den Augen auf der Stirn eingesezt, das folgende Glied klein, kugelförmig, die nun folgenden sechs Glieder sehr klein und dicht zusammengedrängt, dass sie als blosse Ringe erscheinen, die drey lezten Glieder lang und dicker, fast wie bey* Anobium *gebaut, sie machen zusammen zwei Drittheil der ganzen Fühlerlänge aus* ..."]. In addition, Germar gave the length of this specimen as 1^1^/_8_ lines = 2.54 mm. This would be an extremely low value for a species of *Lilioceris*. All extant species are described as being longer than 5 mm. Germar mentioned that his *Criocerina pristina* resembled „*Crioceris testacea* Fabr.”, of which he said it were six times larger – i.e. ca. 1.5 cm. The species Fabricius described as *Crioceris testacea* (Fabricius 1787: 87/88) is currently listed under *Aulacophora indica* (Gmelin, 1790), Galerucinae (Mohamedsaid 2009), and its lectotype is depicted in Lee and Beenen (2015, Figs 42 & 43). We conclude that „*Crioceris pristina*” is actually not a criocerine beetle nor a member of the family Chrysomelidae. Instead, we suggest that it should be better placed as Coleoptera *incertae sedis*. This conclusion leaves *Lilioceris groehni* sp. n. as the first beetle species from Baltic amber that we can classify with certainty as a member of the Coleoptera Chrysomelidae Criocerinae.

## Supplementary Material

XML Treatment for Lilioceris groehni
